# Interviewer effects on non-response propensity in longitudinal surveys: a multilevel modelling approach

**DOI:** 10.1111/rssa.12049

**Published:** 2014-02-19

**Authors:** Rebecca Vassallo, Gabriele B Durrant, Peter W F Smith, Harvey Goldstein

**Affiliations:** University of SouthamptonUK; University of BristolUK

**Keywords:** Area effects, Cross-classified models, Family and Children Study, Interviewer effects, Multiple-membership models

## Abstract

The paper investigates two different multilevel approaches, the multilevel cross-classified and the multiple-membership models, for the analysis of interviewer effects on wave non-response in longitudinal surveys. The models proposed incorporate both interviewer and area effects to account for the non-hierarchical structure, the influence of potentially more than one interviewer across waves and possible confounding of area and interviewer effects arising from the non-random allocation of interviewers across areas. The methods are compared by using a data set: the UK Family and Children Survey.

## Introduction

The rise in survey non-response rates, which has been documented by De Leeuw and De Heer (2002), provides a strong motivation for investigating the causes and factors influencing non-response. Prominent among such studies are those analysing interviewer effects, which aim to reduce non-response at the design stage or during data collection. Studies focusing on interviewer effects are motivated by the understanding that interviewers play an important role in introducing the survey concept, engaging the respondent, addressing any queries and ultimately gaining responses (Groves and Couper, 1998; Hox and de Leeuw, 2002). The studies also acknowledge the possible influence that the research agency can have in minimizing negative interviewer effects through effective policies and management strategies (Sinibaldi *et al*., 2009; Durrant *et al*., 2010).

Although many studies have confirmed the presence of significant interviewer effects on non-response in both cross-sectional (Durrant and Steele, 2009; Blom *et al*., 2010; Durrant *et al*., 2010) and longitudinal surveys (Campanelli and O'Muircheartaigh, 1999; Pickery *et al*., 2001; Pickery and Loosveldt, 2002; Haunberger, 2010), there has been little conclusive or consistent evidence concerning the interviewer attributes that are associated with higher non-response rates. This partly reflects the lack of detailed information on interviewers that is available in many of these studies (Hill and Willis, 2001; Watson, 2003; Nicoletti and Peracchi, 2005).

Possible theoretical arguments for area effects on non-response include similarities in socio-economic and cultural characteristics, perception of privacy, crime and safety as well as environmental factors such as physical accessibility and urbanicity across geographic boundaries (Haunberger, 2010). Studies, such as that carried out by Durrant *et al*. (2010), which assess the effect of both interviewer and area effects on non-response for multistage cluster sample design data, generally seem to indicate that interviewer effects are more important than area effects. The best data for determining the nature of the higher level random effect are data coming from an interpenetrated sample design, where each sampled case is allocated randomly to interviewers irrespectively of the area provenance of the case. A quasi-randomization of cases across interviewers was implemented at the second wave of the British Household Panel Study, where the sample cases that were available for assignment for a specific interviewer were restricted to a geographic pool of two to three primary sampling units (PSUs). Campanelli and O'Muircheartaigh (1999) analysed the significance of area and interviewer random effects on household and individual non-response, refusal and non-contact for these data by using a cross-classified multilevel model. They found no evidence of random effects at the PSU and the geographic pool levels.

For the analysis of interviewer effects, a multilevel modelling approach has been advocated and implemented in various cross-sectional studies (including Hox and de Leeuw (2002), Durrant and Steele (2009), Blom *et al*. (2010) and Durrant *et al*. (2010)). A complicating factor when analysing interviewer effects is that interviewers generally work in a limited geographic area and, to the extent that people from certain areas are more or less likely to co-operate, significant interviewer effects may simply indicate area effects. Few studies, including Campanelli and O'Muircheartaigh (1999) and Durrant *et al*. (2010), have attempted to disentangle interviewer and area effects by specifying a cross-classified multilevel model for multistage cluster sample design data.

For longitudinal surveys, there are added complexities in the analysis of interviewer effects on non-response. Firstly, some cases may be allocated to different interviewers at different waves. Secondly, the response outcome for the same sampled person may vary across waves. However, longitudinal data offer the advantage of obtaining information on both respondents and non-respondents from previous waves when analysing co-operation at a later wave, which is often missing for cross-sectional data. A particular research interest, which is of importance for effective longitudinal survey designs, is the optimal interviewer allocation across waves for the same respondent. This decision must take into consideration the effect of a change of interviewer and the relative effect of interviewers from previous waves on respondent's co-operation at later waves.

There is only limited research on modelling interviewer effects in longitudinal surveys that takes account of the additional complexities, in particular the influence of more than one interviewer across waves. Pickery *et al*. (2001) proposed a multilevel cross-classified logistic model, specifying the effects of the previous and current interviewers as independent effects at the higher level (level 2 in a hierarchical model). More recently Lynn *et al*. (2013) have used a multilevel multiple-membership model (Goldstein, 2011), where the overall interviewer effect is made up of a weighted combination of two wave interviewers. However, the relative benefits of different modelling approaches for the analysis of interviewer effects in longitudinal surveys have not been considered. Also, there has been no study that included various wave interviewer effects and the area effect in one model.

This paper presents a multilevel modelling framework for the analysis of interviewer effects on wave non-response in longitudinal surveys and considers two different multilevel modelling approaches: the multilevel cross-classified and the multiple-membership models. The modelling approach proposed incorporates both interviewer and area effects accounting for the non-hierarchical structure and potential confounding due to a lack of an interpenetrated sample design arising from the non-random allocation of interviewers across areas. An advantage of the multiple-membership modelling approach is that for the estimation of interviewer effects not all cases need to experience a change of interviewer. The separation of the interviewer and area effects is only possible for data having the majority of interviewers work in more than one area and the workload in most areas distributed among at least two interviewers.

The methods are compared by using a data set, with the focus on non-response at a later wave in the life of a longitudinal study, from the UK Family and Children Survey (FACS). In addition to the methodological considerations, the study also makes some substantive contributions. The study considers the relative importance of interviewers across two waves and aims to identify interviewer characteristics that influence non-response behaviour.

The research has various implications for survey practice. The study provides guidance to survey researchers on how best to model the relative influence of several interviewers on non-response across waves. The results may inform decisions how best to allocate interviewers across waves and across cases. The identification of significant interviewer sociodemographic characteristics, work history, personality traits and job attitudes may provide guidelines for more effective interviewer recruitment, training, appraisal and allocation of work.

The remainder of the paper is structured as follows. Section Data describes the data set. Next, the methodology section outlines the relative benefits of the various multilevel modelling approaches. Section Implementation of methods and results applies the models to the specifics of our data set. The final section summarizes key findings and discusses implications for survey practice.

## Data

The FACS gathers information on the health and socio-economic status of households with children in the UK (Lyon *et al*., 2007) and benefits from the availability of rich survey information for both respondents and non-respondents from previous waves of the longitudinal study. A key advantage of the current study is that detailed information on interviewers is linked to the survey data.

The FACS study started in 1999 with a narrow focus on low income families with children and lone parent households, was expanded in 2001 to be representative of all households with children and continues to this date. The study has a two-stage sampling design (Department for Work and Pensions, 2011). First, a sample of 150 PSUs stratified by region and a rural–urban indicator from a total of 2600 postcode sectors (each representing 3000 households on average) listed in the child benefit database were chosen with probability proportional to the number of child benefit records. Secondly, a systematic sample of 100 households with a random start was chosen within each cluster, resulting in 15000 households before any reductions arising from invalidity of address, opt-outs and screening procedures.

Information on interviewers firstly comes from administrative data collected by the survey agency the National Centre for Social Research annually. The administrative data include the identification code of the interviewer who was allocated to each case for all waves, some demographic information, interviewer grade and the years of experience within the Centre. Secondly, the interviewer survey, a postal, self-completion survey administered in May 2008 addressed to all interviewers who had worked for the Centre at some point since the start of 2006, provides rich data on interviewing experience, job expectations and appraisal, flexibility in working hours, personality traits, interpersonal skills and views on the persuasion of reluctant sample members.

For the analysis, the focus is on the non-response behaviour at the later stages of the FACS, here on the last two waves, for which all relevant information was available. These are wave 7 and wave 8, which were conducted in 2005 and 2006 respectively. Co-operation or refusal with regard to the main face-to-face survey interview is investigated. The main outcome of interest is whether or not a person responded to wave 8, conditioning on response to wave 7. This allows detailed information on both the respondents and the non-respondents to wave 8 to be obtained from the previous wave. The analysis is conditional on contact being made with the household, similarly to the work by Watson and Wooden (2006), Blom *et al*. (2010) and Durrant *et al*. (2010). The data set includes only a small number of non-contact cases, amounting to 2.6% of cases in wave 8, compared with 8.5% of cases resulting in refusals or unproductive interviews. It should be noted that, in analysing refusal for wave 8 of the FACS (2006), the interviewer variables that were obtained from the interviewer survey (2008) represent the interviewer characteristics approximately 2 years later. However, this discrepancy is not believed to have a significant effect on the results since most information from the interviewer survey that is used here, e.g. information on behaviours and personality traits, is assumed to be relatively stable over time (Sinibaldi *et al*., 2009). Other information that would be expected to change, such as age and interviewers' experience, is taken from the annually collected administrative data on interviewers.

The initial data set includes the wave 8 cases that had participated in wave 7. A wave number represents the number of survey episodes since its inception in 1999. As there are varying initial waves and numbers of interviews for different cases considered in the data set, participation history variables are included as controls in the multilevel models. Cases with missing values for interviewer variables that were used in the final model have been dropped. Unit non-response in the interviewer survey is controlled for by including interviewer variables from the interviewer administrative data that are available for all interviewers, such as gender, experience and grade. The final analysis sample includes 5932 cases pertaining to 307 wave 7 interviewers, 275 wave 8 interviewers and 150 PSUs. About 68.3% of cases changed their interviewer between waves 7 and 8, such that 73.1% of wave 8 interviewers had cases that were associated with different interviewers across the two waves. A change in interviewer allocation may arise because of a move of the household or changes in the interviewer's responsibility and workload, or the interviewer may have stopped working for the survey agency. For wave 8 there are no PSUs in which only one interviewer was allocated work, and approximately 82% of interviewers were allocated households from at least two different PSUs. This results in a cross-classification of interviewers by PSUs.

## Methodology

### Methods for analysing clustered data: a multilevel modelling framework

Most survey data are hierarchical in nature owing to, for example, a multistage sampling procedure or the allocation of cases to interviewers for face-to-face surveys. Clustering of non-response at the higher level may reflect various unmeasured factors, including an interviewer's demographic characteristics, skills, attitudes, behaviour and personality traits, or the cultural and socio-economic nature of the area sampled. Consequently, the violation of the assumption of independence of observations forbids the use of standard analytical techniques that do not correct for increased standard errors, which can therefore result in incorrect inference (Snijders and Bosker, 1999). Both disaggregated and aggregated approaches can adjust the standard error estimates to account for the dependence in the clustered data. Whereas in aggregated methods design variables are only implicitly accounted for by averaging the effect of other explanatory variables over the population distribution of these design variables, in disaggregated methods design variables may be treated as scientifically relevant and explicitly incorporated in the model. A disaggregated approach is therefore the preferred method here since the area and interviewer clusters are of scientific interest in the study of non-response and may directly affect the outcome variable. Moreover, a disaggregated approach allows the effect of the population indicators (design variables) on the outcome variable survey non-response to be quantified.

Various analytical techniques for the analysis of interviewer effects have been developed (Von Sanden, 2004). Multilevel modelling, which is a disaggregated approach, has become a popular method of choice in research analysing interviewer effects (Hox and de Leeuw, 2002; Pickery and Loosveldt, 2002; Durrant and Steele, 2009; Blom *et al*., 2010; Durrant *et al*., 2010; Haunberger, 2010; Lynn *et al*., 2013). Advantages of multilevel models include their ability to account for different hierarchical structures of survey data and the treatment of clustering as an integral aspect of the analysis rather than a nuisance simply to be accounted for. In particular, multilevel models allow the investigation of substantive research questions that go beyond the scope of standard approaches, such as the possibility of analysing the amount of total variation that is attributable to interviewer effects. Moreover, multilevel models allow the estimation of the relative effect of different wave interviewers on current wave non-response.

### Accounting for area and multiple-interviewer effects across waves

A complicating factor when analysing interviewer effects is their potential confounding with areas (PSUs). For many face-to-face surveys an interviewer will work almost exclusively in a limited geographic area. Therefore, variation in the probability of refusal by interviewer may simply reflect area differences in the geographic propensity to co-operate in survey requests. Very few studies exist that have been able to use an interpenetrated sample design (Campanelli and O'Muircheartaigh, 1999; Schnell and Kreuter, 2005), enabling, to some extent, a separation of interviewer and PSU effects. A fully random allocation of interviewers to households for face-to-face surveys would be very costly and therefore practically very difficult. In some studies, as here, a complete confounding of interviewers and areas is avoided, since interviewers and areas are partially interpenetrated (Von Sanden, 2004; Durrant *et al*., 2010). This means that interviewers are not fully nested within areas, as one interviewer may work in more than one PSU, and cases in one PSU may be designated to more than one interviewer. With a data structure showing partial interpenetration, a multilevel cross-classified model specification which considers both interviewer and area random terms can allow for a distinction between interviewer and area effects (Goldstein (2011), chapter 12).

The easiest way of accounting for the influence of interviewers in a longitudinal survey is to consider only the current wave interviewer. However, as shown in Goldstein (2011), chapter 13, assigning a case to just one level 2 unit (in this case interviewers) when in fact the case has multiple memberships, i.e. it belongs to more than one higher level unit, will lead to an underestimation of the higher level variance. It may be hypothesized that more than one interviewer, and potentially all interviewers who are associated with each case, have an influence on the non-response outcome. In this paper two different approaches to specifying the various wave interviewer random effects are considered. One is to specify these effects as cross-classified (Goldstein, 1994), as was done in the study by Pickery *et al*. (2001), thereby assuming that the interviewer effects for each wave are independent. However, in the context of (at least some) interviewer continuity across waves, the tenability of this assumption is questionable. An alternative approach involves the use of a multilevel multiple-membership model (Goldstein, 2011). To our knowledge, Lynn *et al*. (2013) is currently the only application of this kind in this area. This specification takes account of all the distinct interviewers to whom each case was allocated across the various waves considered. Such models allow the effect of all distinct interviewers who were associated with a specific case to be incorporated in the model by attributing a weight to each interviewer effect; together these sum to a weight of 1, such that the estimated effect becomes a weighted average of all interviewers.

Controlling for both complicating factors simultaneously, i.e. the confounding of area and interviewer effects and the influence of multiple interviewers per household across waves, leads to two possible specifications. Under the assumption of independent interviewer effects, a cross-classified model with various distinct random effects (one area effect and an interviewer effect for every wave) is obtained. Under the multiple-interviewer membership assumption, a model specifying an area random effect cross-classified with the interviewer multiple membership is obtained. This is referred to as a multiple-membership multiple-classification (MMMC) model.

### Model specifications

Let *y*_*i*(**j***s*)_ denote the dependent binary variable of interest, indicating whether individual *i*, interviewed by interviewers **j**=(*j*_1_,…,*j*_*n*_) in waves *k*=1,…,*n* and living in PSU *s*, refused to participate at wave *n*. Contact at wave *n* and response at wave *n*−1 are assumed. The general forms of the two most comprehensive multilevel logistic model specifications considered are presented below. Although both models include a cross-classified area effect, the first model considers the various wave interviewers as cross-classified, whereas the second model considers a multiple membership for the interviewer allocation.

The general form of the cross-classified multilevel logistic model is

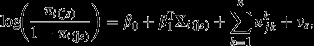
1where *π*_*i*(**j***s*)_=Pr(*y*_*i*(**j***s*)_=1) is the probability that individual *i* refuses to participate at wave *n*. The parameter *β*_0_ represents the overall intercept in the linear relationship between the log-odds of refusal and predictor variables specified in the model, **X**_*i*(**j***s*)_. The vector ***β***_1_ contains the coefficients for the explanatory variables in the model. The parameters 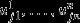 and *v*_*s*_ represent the random effects for each wave interviewers *j*_1_,…,*j*_*n*_, and the individual's area of residence respectively, which are assumed to follow a normal distribution with variances 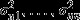 and 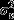. The model includes an independent random effect for each wave interviewer considered.

The general form of the MMMC approach is

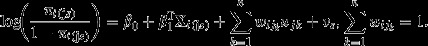
2

The outcome variable and the fixed effects for the MMMC model have the same meaning and interpretation as those for the cross-classified model. The MMMC model includes one overall interviewer level random effect *u*_**j**_, which, after combining weights for the same interviewer, is a weighted average of *m* distinct interviewers associated with a case in the *k* waves considered. In contrast, for the cross-classified multilevel model there are *n* unique interviewer effect distributions: one for each wave considered. The MMMC model also includes an area effect *v*_*s*_ cross-classified with the interviewer effect. Whereas there is only one common distribution for all interviewer effects from various waves, each individual has *m* distinct interviewer effects. These distinct interviewer effects and the individual's area residence effect *v*_*s*_ are assumed to follow independent normal distributions with variances 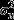 and 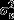.

The term 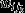 represents the combined weights. Cases who are allocated to the same interviewer across all waves have a weight of 1 for wave *n* and a weight of 0 for all previous waves, whereas cases experiencing a change of interviewer have at least two non-zero weights summing to 1. The number of non-zero weights is thus equal to the number of distinct interviewers *m* who were associated with that particular case. Therefore, each case may have from 1 to *n* distinct interviewers.

The choice of which weights to apply can be empirically or theoretically based. There are two different justifiable theoretical arguments for the choice of weights. One possible argument is to allocate weights that are proportional to the number of waves that the interviewer was allocated the specific case, reflecting the amount of time covered by each interviewer. Alternatively, one may argue that although the decision to take part in the current wave will be somewhat influenced by the experience in the prior waves, the current wave experience has a greater effect, since the current wave interviewer has the possibility to interact actively and to address any hesitancies, and should therefore be given a greater weight. However, for both options there is still some arbitrariness in the choice of weights. The weight profile can vary by both the number of interviewers and the sequence of interviewer changes. Alternatively, the choice of weights may be guided by an empirical assessment, as proposed in Goldstein (2011) and advocated in Lynn *et al*. (2013), using the deviance information criterion (DIC) value for alternative models including various weight specifications. The DIC is a Bayesian measure of model fit which penalizes for model complexity, therefore allowing non-nested models to be compared (Spiegelhalter *et al*., 2002).

### Model estimation and modelling strategy

Both cross-classified and multiple-membership models can be estimated by using Markov chain Monte Carlo methods with the quasi-likelihood estimates as starting values (Goldstein and Rasbash, 1996; Goldstein, 2011), as implemented in the MLwiN software (Browne, 2012). These methods have been shown to produce improved estimates over first-order marginal quasi-likelihood and second-order penalized quasi-likelihood in terms of frequentist unbiasedness (Browne, 1998).

A forward selection strategy is used. The first step is the specification of the random-effects structure, excluding any covariates in the model. Then the inclusion of fixed effects in the model is considered. To understand the random terms better and to identify whether the more complicated models are an improvement on simpler models, multilevel models including only one random effect are fitted first, and then more random terms are added until the two most complicated models are obtained. The DIC is used for model comparison, with a smaller DIC indicating a better fit. Significance testing for random effects is based on the Wald test. Since variances cannot be negative one-sided *p*-values are used (Snijders and Bosker, 1999).

## Implementation of methods and results

### Model specification for the Family and Children Survey example data set

The methods proposed for the analysis of interviewer effects in a longitudinal survey are applied to the FACS data. Owing to the changing nature of the sample across waves, and the high number of missing data for previous waves, reflecting administrative failures in the registration of case allocation to interviewers, accurate and complete data for the FACS are available for only the last two waves. Therefore, the focus is on the last two waves, and models accounting only for both the interviewer from the current wave (wave 8) and the interviewer from the previous wave (wave 7) are considered. The respondent area identifier for the FACS data set is specified at wave 7. The area effect in this context is considered mainly to be the aggregate effect of unmeasured socio-economic and cultural determinants of non-response across communities having similar backgrounds. A new area of residence, following a household move, may alter these household's characteristics, but this will only realistically happen over time. Consequently, a household move between waves should not bring an immediate change in the ‘area’ effect for that household.

The two most comprehensive models considered for this data set are a cross-classified model with three distinct random effects, one for area, one for the wave 7 interviewer and one for the wave 8 interviewer, and an MMMC model specifying an area random effect cross-classified with the interviewer multiple membership:

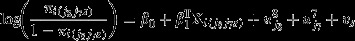
3and

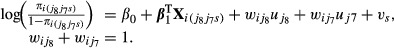
4In the MMMC model, the overall interviewer random effect is a weighted average effect of the two interviewers whom each individual is allocated at wave 8 and wave 7. These weights are represented by 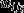 and 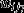. Whereas cases who are allocated to the same interviewer across both waves are given a combined weight of 1 for 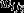 and a weight of 0 for 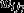, cases experiencing a change of interviewer have two non-zero weights (identical for all cases) summing to 1. The way that the models are set up in MLwiN requires this combined weight specification, rather than the equivalent theoretical specification of equal 0.5 weights, for cases who are associated with only one higher level unit (Browne, 2012). Different pairs of non-zero weights are considered, varying by 0.1, from weights of (0.9, 0.1) to (0.1, 0.9). The weight profile corresponding to the lowest DIC is chosen. Unless otherwise stated, all models are estimated by using Markov chain Monte Carlo estimation in MLwiN version 2.20 with default priors, a burn-in length of 5000 and 500000 iterations. Here Markov chain Monte Carlo estimation is used simply to maximize the likelihood for the variance parameters, and it has been shown that using the default priors integrated in MLwiN gives similar estimates to maximum likelihood estimation (Browne (2012), chapter 6).

Once an appropriate random-structure specification has been identified, groups of explanatory variables are considered for inclusion in the following order: participation history, household, area and interviewer variables. Descriptive statistics for area and interviewer variables are included in on-line ‘supporting information’. Potential predictors are chosen on theoretical grounds and a review of significant predictors in the literature. Once all variables pertaining to a particular group have been included, their significance is assessed again and variables with a *p*-value less than 0.05 are retained at subsequent steps when including variables from other groups irrespectively of their *p*-value thereafter. In this analysis the interviewer's gender and a grade or experience variable, predictive of non-response in the interviewer survey, are included as control variables in the models irrespectively of their significance.

### Random-effects specification

First, multilevel models including only one random effect at a time are explored. From Table1 it can be seen that all models indicate significant results for the higher level random effects. The model including the wave 8 interviewer random effect (model 3) has the best fit. The ‘DIC change’ column gives the difference in DIC for the model being considered and model 3. The model with the wave 7 interviewer random effect (model 2) has a small increase in DIC, indicating a slightly worse fit compared with model 3, whereas the model with the area random effect (model 1) has a large increase in DIC.

**Table 1:** Variance estimates and DIC values for various specifications of two-level models

Model	Random terms in the model	Variance (standard error)	DIC	DIC change
0	None		4197.09	46.45
1	PSU	0.122 (0.051)†	4178.81	28.17
2	Interviewer 7	0.233 (0.065)†	4155.98	5.34
3	Interviewer 8	0.273 (0.077)†	4150.64	

† Significant at the 1% level.

Multilevel cross-classified models including only one of the interviewer random effects in addition to the area effect are considered next (models 4 and 5 in Table2). For both models, the area random variance is no longer significant. The DIC values for models 4 and 5 are only slightly lower than those obtained for the equivalent models 2 and 3 controlling only for the respective interviewer effect, indicating that these cross-classified models do not offer a noticeable improvement over the simpler two-level models.

**Table 2:** Variance estimates and DIC values for various specifications of cross-classified models

Model	Random terms in the model	Variance (standard error)	DIC	DIC change
4	Interviewer 7 and PSU	0.210 (0.068)†;	4153.77	3.13
	cross-classification	0.048 (0.044)		
5	Interviewer 8 and PSU	0.247 (0.079)†	4149.79	−0.85
	cross-classification	0.047 (0.044)		
6	Interviewer 7 and interviewer	0.139 (0.080)‡	4146.27	−4.37
	8 cross-classification	0.167 (0.095)‡		
7	Interviewer 7, interviewer	Did not converge		
	8 and PSU cross-classification			

† Significant at the 1% level.

‡ Significant at the 5% level.

Despite obtaining a lower DIC value than model 3, a cross-classified model controlling for both interviewers at wave 7 and 8 but excluding area effects (model 6) yields numerically unstable results. Stability is reached only once the Markov chain Monte Carlo chain length is increased to 5 million iterations, at which point both effects are only just significant. This instability suggests that the two interviewer random effects are near non-identifiable. The cross-classified model with all three random effects (model 7) did not converge. The unstable results for the cross-classified interviewer random effects converge, which may indicate that the assumption of independent interviewer effects is erroneous, resulting in a misspecification of the interviewer level structure.

As an alternative, multiple-membership models are explored using a range of weights (Table3). The weights that are allocated for cases experiencing a change of interviewer are specified in Table3. The results show that the multiple-membership models that give a weight of at least 0.8 to wave 8 interviewers provide a better model (models 15 and 16). These results support the hypothesis that the wave 8 interviewer has the greatest influence on the current wave non-response. The next best fitting models are obtained for a high wave 7 weight (models 13 and 14); these perform better than models allocating moderate weights (approaching an equal share) to both wave interviewers (models 8, 9 and 10). This would seem to suggest that there is not much difference between the wave 7 and wave 8 interviewer effects.

**Table 3:** Multiple-membership and MMMC models

Model	Random terms in the model	Wave 8, wave 7 weights	Variance (standard error)	DIC	DIC change
8	Multiple-interviewer membership	0.4, 0.6	0.278 (0.086)†	4159.08	8.44
9	Multiple-interviewer membership	0.5, 0.5	0.287 (0.087)†	4159.03	8.39
10	Multiple-interviewer membership	0.6, 0.4	0.288 (0.090)†	4158.75	8.11
11	Multiple-interviewer membership	0.3, 0.7	0.272 (0.082)†	4158.33	7.69
12	Multiple-interviewer membership	0.7, 0.3	0.291 (0.090)†	4157.41	6.77
13	Multiple-interviewer membership	0.2, 0.8	0.262 (0.076)†	4157.38	6.74
14	Multiple-interviewer membership	0.1, 0.9	0.250 (0.074)†	4155.85	5.21
15	Multiple-interviewer membership	0.8, 0.2	0.288 (0.085)†	4155.36	4.72
16	Multiple-interviewer membership	0.9, 0.1	0.282 (0.081)†	4153.12	2.48
17	Multiple-interviewer membership	0.9, 0.1	0.252 (0.084)†;	4151.62	0.98
	and PSU cross-classification		0.049 (0.049)		

† Significant at the 1% level.

The MMMC model (model 17) with the preferred weight specification (0.9, 0.1) shows a slight improvement in the model fit compared with the multiple-membership model that does not account for the area cross-classification (model 16). However, the area random effect in this model is not significant. The consistent increase in the DIC for the MMMC models indicate that, for our application, the multiple-membership models do not provide a significant improvement over the simpler two-level model, which accounts only for the current wave interviewer (wave 8).

In conclusion, for this application, simply controlling for one wave interviewer may be sufficient. The area random effect is shown across all models to be negligible once the interviewer random effect has been controlled for, warranting its exclusion from the model. The two-level models indicate that the wave 8 interviewer random effect (model 3) produces a slightly better model fit than the model with wave 7 interviewer effect (model 2), and also that it explains a larger proportion of the total variance. Consequently, the final model accounts for only the wave 8 interviewer clustering.

### Discussion of the final model—random effects

Table4 presents estimates of the interviewer 8 random effect as groups of explanatory variables are added to model 3, for the final data set of 5932 cases. Each row therefore represents a new model, specified as the previous row model with the addition of new variables (as specified in the first column of Table4). Also included in Table4 are the percentage reduction in variance compared with the initial variance obtained in the null model (the column headed ‘(a) % reduction’) or compared with the variance that is obtained in the preceding model (the column headed ‘(b) % reduction) and the DIC. VPC measures the proportion of total variance attributable to differences between interviewers. For any random-intercept model, VPC is

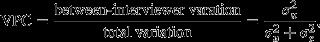
5where 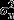 is the interviewer level and 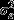, the individual level variance, is specified as 3.29 by using the threshold specification of a logistic model (Snijders and Bosker, 1999; Goldstein, 2011). The DIC values indicate a better model fit at each step of the model building exercise, as more significant fixed effects are included in the model.

**Table 4:** Estimates of the interviewer 8 random effect as groups of explanatory variables are added

Fixed effects parameter (number of parameters)	Variance (standard error)	(a) % reduction	(b) % reduction	VPC	Model DIC
None	0.279 (0.088)†	—	—	7.8	3397.7
Added interviewer change (1)	0.198 (0.081)†	29.0	29.0	5.7	3385.6
Added participation history (2)	0.179 (0.078)‡	6.8	9.6	5.2	3363.6
Added respondent or household characteristics (12)	0.158 (0.083)‡	7.5	11.7	4.6	3363.6
Added interviewer 8 gender and grade or experience (7)	0.105 (0.076)	19.0	33.50	3.1	3327.2
Added interviewer 8 work history (4)	0.090 (0.070)	5.4	14.3	2.7	3316.4
Added interviewer 8 personality trait (3)	0.057 (0.057)	11.8	36.7	1.7	3309.8
Added interviewer 8 skills (4)	0.044 (0.049)	4.7	22.8	1.3	3303.0

† Significant at the 1% level.

‡ Significant at the 5% level.

The interviewer random effect remains significant until the inclusion of the interviewer administrative variables: gender and interviewing grade or experience. The variables that are included in the model explain all of the interviewer random effect. This supports the findings by Campanelli and O'Muircheartaigh (1999) who found interviewer effects to be no longer significant once fixed effects had been controlled for. For the first model, not including any fixed effects, the interviewer variance accounts for around 7.8% (0.279/(0.279 + 3.29)× 100) of the total variation in refusal at wave 8. Once variables for interviewer change, participation history, household level, interviewer experience and grade, work history, personality traits and skills have been controlled for, the variance partitioning coefficient is reduced to 1.3%. Although the interviewer variance also decreases slightly when including the 12 household level fixed effects, the more substantial decreases come from the interviewer level variables.

Before deciding on the final specification, multiple-membership models with different weight specifications are revisited to evaluate their performance in comparison with the simpler two-level model. However, no noticeable differences in the DIC values are found, implying that, for this application, a simpler two-level model is indeed sufficient, even after the inclusion of explanatory variables. Including an area random effect instead of the interviewer random effect, while maintaining all fixed effects, results in a higher DIC value and a non-significant area effect.

### Discussion of the final model—fixed effects

Table5 presents the estimated coefficients of the final multilevel logistic regression model. Household level characteristics are entered as control variables but are not discussed here. The variables describing the geographical area of the household, such as the indicator for the UK regions, the London indicator and various respondent neighbourhood perception variables are found not to be significant after controlling for other household level variables, confirming similar findings in Durrant *et al*. (2010). This result supports the conclusion that, after controlling for household and interviewer effects, area effects are negligible. The following discussion focuses on the effects of interviewer level variables.

**Table 5:** Estimated coefficients for the final multilevel logistic model

Variable [reference category]	Category	β	Standard error	β/(standard error)	p-value
Interviewer change {same}	Change	0.409	0.110	3.718	0.000
*Participation history variables*					
First wave for respondent {wave 7}	Wave 1–wave 4	−0.892	0.145	−6.152	0.000
	Wave 5–wave 6	−0.443	0.163	−2.718	
*Respondent or household variables*					
Ethnicity {non-white and missing}	White	−0.524	0.155	−3.381	0.001
Any vocational or academic qualifications {yes}	No	0.289	0.144	2.007	0.044
Age of youngest child	0–4 years	−0.548	0.170	−3.224	0.004
{no dependent children and	5–10 years	−0.441	0.171	−2.579	
16–18-year-olds}	11–15 years	−0.189	0.175	−1.080	
Heating problems in the dwelling {no and ‘don’t know’}	Yes	0.266	0.217	1.226	0.225
Gender {female}	Male	−1.428	0.554	−2.578	0.010
Accommodation type {detached house}	Semi-detached house	−0.280	0.125	−2.240	0.198
	Terraced house	−0.264	0.137	−1.927	
	Flat or maisonette—purposebuilt and other	−0.159	0.211	−0.754	
	Flat or maisonette—conversion	−0.504	0.523	−0.964	
Household size	Household size	0.086	0.046	1.870	0.056
*Interviewer 8 administrative variables*					
Gender {female}	Male	0.094	0.111	0.847	0.391
Grade experience {grade R; S, 5 or more years experience; T (highest grade)}	Grade A (lowest grade); B	1.117	0.278	4.018	0.000
	Grade C, 0–4 years experience	1.027	0.212	4.844	
	Grade C, 5 or more yearsexperience	0.495	1.640	0.234	
	Grade D, 0–4 years experience	0.812	2.252	0.254	
	Grade D, 5 or more yearsexperience	0.530	1.699	0.252	
	Grade S, 0–4 years experience	0.701	2.016	0.286	
*Interviewer 8 work history, time availability, attitudes towards refusal, work priorities, satisfaction with*					
*job variables*					
Interviewing work history—work status with anothersurvey agency and experience with other (phone,marketing) survey interviewing {never worked foranother survey agency}	Working with another agencyat time of survey and doneother survey interviewing	−0.208	0.174	−1.195	0.000
	Worked with another agencybefore January 1st, 2006,and done other survey interviewing	0.436	0.131	3.328	
	Working with another agencyat time of survey and neverdone other survey interviewing	−0.610	0.296	−2.061	
	Worked with another agencybefore January 1st, 2006,and never done other surveyinterviewing	0.241	0.185	1.303	
*Interviewer 8 personality traits variables*					
Worries a lot 1 {does not applyto me at all)}	2, 3	0.582	0.204	2.853	0.002
	4	0.298	0.220	1.355	
	5, 6, 7 (applies perfectly to me)	0.690	0.213	3.239	
*Interviewer 8 skills traits variables*					
Express myself easily	4	0.342	0.291	1.175	0.011
{1 (does not apply to	5, 6, 7 (applies perfectly to me)	0.638	0.251	2.542	
me at all), 2, 3}					
Can't help but look upsetwhen something bad	1 (does not apply to me at all), 2	−0.28	0.17	−1.709	0.053
happens {6, 7 (applies perfectly to me)}	3, 4, 5	−0.33	0.14	−2.416	

In agreement with previous observational studies (Schatteman, 2000; Watson and Wooden, 2006; Haunberger, 2010), a change of interviewer between waves is positively associated with refusal. However, a causal relationship cannot be inferred as a change of interviewer may reflect a respondent's move or the resignation of an interviewer rather than a random allocation (Hill and Willis, 2001). Compared with the highest group, all the other categories of the grade or experience variable show a significantly higher propensity of refusal at the 5% level. As hypothesized, interviewers in the lower experience group for both grades C and D have higher odds of refusal than those interviewers in the higher experience group for the same grade. The difference between these groups is, however, only significant for grade C. The positive effect of grade and experience is a consistent effect that has been confirmed across various studies (Campanelli *et al*., 1997; Hox and de Leeuw, 2002; Pickery and Loosveldt, 2002; Hansen, 2006; Durrant *et al*., 2010) and may indicate either improved performance over time and as one moves up in the company hierarchy or a selection effect with better interviewers remaining in the industry and being promoted.

The work history variable shows that having previously worked with another survey agency and experience in other types of interviewing have a negative effect on individual level co-operation. The negative effect of work experience in different interviewing modes or research areas suggests that face-to-face interviewing requires specific tactics and skills, and that exposure to techniques that are suitable for other types of interviewing may hamper performance. The results indicate that interviewers working with another survey agency at the time of the survey performed better than those who had previous experience with another survey agency and better than those with no such experience. This result may indicate that interviewers who commit most of their paid working hours undertaking interviewing work for various survey agencies perform best. The explanation for the negative effect of previous work with other survey agencies is somewhat unclear and more data on interviewing work history is required to explore this relationship further. Possibly, job tenure in interviewing work shows commitment and skill in one's work.

Personality traits and interviewer skills have been hypothesized to play a role in explaining interviewers’ performance (Weinhardt and Kreuter, 2011). Here, only one personality item was retained in the final model. This item indicates that respondents who are allocated interviewers with a low or high self-rating of neuroticism in terms of worrying tendencies are more likely to refuse participation than those respondents who are allocated to interviewers who assert that this item does not describe them at all. In contrast, interviewers showing a moderate score of 4 are not significantly different from the reference category at the 5% level. Two skills items were retained in the final model: ‘can’t help but look upset when something bad happens’ and ‘express myself easily’. Contrary to expectations, respondents who were approached by interviewers rating themselves as highly capable of expressing themselves with ease (5, 6, 7) are significantly more likely to refuse than those who were allocated to interviewers who rated themselves poorly (1, 2, 3) on this skill, whereas there is no significant difference for those interviewers with moderate scores (4). This result possibly indicates that interviewers who are less complacent about their ability to convey the survey message, who have greater awareness of the way that they portray themselves and who make a conscious effort to communicate effectively achieve higher response rates. Sample cases who were allocated to interviewers who perceive themselves as never allowing others to notice that they are upset when something goes wrong are less likely to obtain respondent level refusals than interviewers who recognize that they are very likely to show such feelings. This result might highlight that interviewers who do not become flustered or defeatist if a sampled person shows scepticism or hesitancy have more chance of success.

Of substantive interest is the recurrent pattern for personality and skills items, where interviewers providing moderate answers scored generally better than those providing extreme values. This may indicate that the most confident interviewers do not necessarily perform best on the job. It may also be plausible that, whereas interviewers who are confident in their performance may have been more likely to tick moderate scores on the traits items in the interviewer questionnaire, others were more subject to social desirability bias and tended to overrate their personality disposition and skills for the job.

## Discussion

As demonstrated in this paper, cross-classified and multiple-membership multilevel models provide a flexible class of models for the analysis of interview effects on non-response in longitudinal surveys. A cross-classified model can distinguish area and interviewer effects in the case of partial interpenetration, which is sometimes present in surveys. Cross-classified and multiple-membership specifications can account for the various interviewers who were allocated to a particular case across waves. The analysis of wave 8 non-response for the UK FACS serves as an example to assess the methods proposed. The main results from this application are as follows.

- The findings for the multiple-membership models indicate that the model allocating the highest weight to the current wave interviewer fits best.
- For this example, the multiple-membership model does not seem to provide an improvement on the simpler, two-level model which accounts only for the current wave interviewer random effect.
- Area effects are not significant after controlling for interviewer and household level effects in a cross-classified model.
- The unstable estimates that were obtained from the cross-classified model controlling for both interviewers at wave 7 and 8 but excluding area effects suggest that the assumption of independent interviewer effects is erroneous.
- The substantive findings confirm that interviewer experience, grade and continuity are significant predictors of non-response, whereas the role of interviewer personality traits is not clear or coherent.

These findings indicate that for the later stages of a longitudinal survey the current wave interviewer seems to have the greatest influence on current wave non-response. They are in contrast with earlier findings by Pickery and Loosveldt (2002) who reported that the first interviewer has the greatest influence. However, they investigated interviewer effects at the beginning of a longitudinal study, analysing wave 1 and 2 interviewers, and used a cross-classified multilevel model.

Area effects are not significant after controlling for interviewer and household level effects in a cross-classified model, supporting findings by Campanelli and O'Muircheartaigh (1999) and Durrant *et al*. (2010). The non-significance of the cross-classified area effect in comparison with the significant area effect in a two-level hierarchical model suggests either that there is insufficient interpenetration to disentangle the two random effects correctly, or that area effects are simply aggregated interviewer effects. Alternatively, the physical, social and cultural spatial divisions that are related to non-response patterns may not match the PSU classification. The possibility of a significant area effect for a different area classification cannot be completely ruled out, raising questions on the validity of the results obtained in the case of an omitted crossed factor (Luo and Kwok, 2009).

The substantive results confirm previous findings on the positive relationship between wave participation and interviewer experience, grade and continuity variables, highlighting for example the importance of retaining experienced interviewers within the agency. The non-random nature of interviewer change in observational studies, however, hinders the interpretation of the effect of interviewer continuity on non-response (for an investigation of this effect by using experimental data see Lynn *et al*. (2013)). The current study also sheds light on the need for further data on the work history of interviewers, as results indicate that experience in other interviewing modes and survey areas may be detrimental in obtaining co-operation in face-to-face interviewing in social surveys. The results do not provide much support for the hypothesis that interviewers' personality traits are important predictors of wave non-response. Despite being categorized as skills items, some of these items seem to be representing very specific personality characteristics rather than learnable behaviours. The non-significance of some of these variables may, however, simply reflect an inadequate construct of personality and skill, or possibly a conscious decision taken by some interviewers to answer the questions in a favourable way, leading to distorted personality and skills assessments. Even if there is a relationship between a household's propensity to respond and the interviewer's personality, it may be too weak or complex to identify and may therefore be of limited use in guiding the recruitment and training of interviewers.

Further research has included simulation studies investigating how well cross-classified or multiple-membership multilevel models estimate the variance components. Difference scenarios have been explored, including different levels of area and interviewer cross-classification and different proportions of interviewer continuity.
