# Supplementary material for: Interviewer effects on non-response propensity in longitudinal surveys: a multilevel modelling approach
Source: J R Stat Soc Ser A Stat Soc. 2014 Feb 19;178(1):83–99. doi: 10.1111/rssa.12049 (PMC4282753; doi:10.1111/rssa.12049)
Supplement: Supplementary file 1 [file rssa0178-0083-sd1.pdf]

Table 1: Frequency Distribution of the Response Outcome by Area Categorical Variables for the Individual-Level Data

Category	Statistic	Productive Interview	Refusals	Total
Government Office Region				
North East	Count	349	28	377
	%	92.6%	7.4%	100.0%
North West & Merseyside	Count	635	74	709
	%	89.6%	10.4%	100.0%
Yorkshire & Humber	Count	547	44	591
	%	92.6%	7.4%	100.0%
East Midlands	Count	470	33	503
	%	93.4%	6.6%	100.0%
West Midlands	Count	587	49	636
	%	92.3%	7.7%	100.0%
South West	Count	535	47	582
	%	91.9%	8.1%	100.0%
Eastern	Count	439	40	479
	%	91.6%	8.4%	100.0%
London	Count	403	52	455
	%	88.6%	11.4%	100.0%
South East	Count	633	69	702
	%	90.2%	9.8%	100.0%
Wales	Count	360	35	395
	%	91.1%	8.9%	100.0%
Scotland	Count	473	30	503
	%	94.0%	6.0%	100.0%
London Indicator				
Not London	Count	5028	449	5477
	%	91.8%	8.2%	100.0%
London	Count	403	52	455
	%	88.6%	11.4%	100.0%
How much of a problem are vandalism, graffiti and other deliberate damage to property or vehicles?				
Very big problem/Fairly big problem	Count	945	77	1022
	%	92.5%	7.5%	100.0%
Not a very big problem	Count	1747	142	1889
	%	92.5%	7.5%	100.0%
Not a problem at all/It happens but it is not a problem/Don't know	Count	2739	282	3021
	%	90.7%	9.3%	100.0%
How much of a problem is rubbish or litter lying around?				
Very big problem/Fairly big problem	Count	988	89	1077
	%	91.7%	8.3%	100.0%
Not a very big problem	Count	1943	158	2101
	%	92.5%	7.5%	100.0%
Not a problem at all/It happens but it is not a problem/Don't know	Count	2500	254	2754
	%	90.8%	9.2%	100.0%

How much of a problem are teenagers hanging around on the street?				
Very big problem/Fairly big problem	Count	1331	107	1438
	%	92.6%	7.4%	100.0%
Not a very big problem	Count	1518	120	1638
	%	92.7%	7.3%	100.0%
Not a problem at all/It happens but it is not a problem/Don't know	Count	2582	274	2856
	%	90.4%	9.6%	100.0%
How much of a problem are troublesome neighbours?				
Very big problem/Fairly big problem	Count	386	35	421
	%	91.7%	8.3%	100.0%
Not a very big problem	Count	682	57	739
	%	92.3%	7.7%	100.0%
Not a problem at all/It happens but it is not a problem/Don't know	Count	4363	409	4772
	%	91.4%	8.6%	100.0%
How much of a problem is people being attacked or harassed because of their skin colour, religion or ethnic origin?				
Very big problem/Fairly big problem	Count	167	19	186
	%	89.8%	10.2%	100.0%
Not a very big problem	Count	546	34	580
	%	94.1%	5.9%	100.0%
Not a problem at all/It happens but it is not a problem/Don't know	Count	4718	448	5166
	%	91.3%	8.7%	100.0%
How much of a problem are people being drunk or rowdy in public places?				
Very big problem/Fairly big problem	Count	666	56	722
	%	92.2%	7.8%	100.0%
Not a very big problem	Count	1207	103	1310
	%	92.1%	7.9%	100.0%
Not a problem at all/It happens but it is not a problem/Don't know	Count	3558	342	3900
	%	91.2%	8.8%	100.0%
How much of a problem are people using or dealing drugs?				
Very big problem/Fairly big problem	Count	823	67	890
	%	92.5%	7.5%	100.0%
Not a very big problem	Count	776	64	840
	%	92.4%	7.6%	100.0%
Not a problem at all/It happens but it is not a problem/Don't know	Count	3832	370	4202
	%	91.2%	8.8%	100.0%
Likelihood that purse/wallet is returned to you if found in the street by someone living in your neighbourhood				
...very likely, quite likely,	Count	3052	292	3344
	%	91.3%	8.7%	100.0%
not very likely,	Count	1281	109	1390
	%	92.2%	7.8%	100.0%
or not at all likely? Don't Know	Count	1098	100	1198
	%	91.7%	8.3%	100.0%

Table 2: Frequency Distribution of the Response Outcome by Interviewer Categorical Variables for the Individual-Level Data

Category	Statistic	Productive Interview	Refusals	Total
Wave 8 Interviewer Grade/ Experience				
A; B	Count	354	42	396
	%	89.4%	10.6%	100.0%
C, 0-4 yrs	Count	1919	229	2148
	%	89.3%	10.7%	100.0%
C, 5yrs+	Count	1049	68	1117
	%	93.9%	6.1%	100.0%
D, 0-4 yrs	Count	472	52	524
	%	90.1%	9.9%	100.0%
D, 5yrs+	Count	533	48	581
	%	91.7%	8.3%	100.0%
S, 0-4 yrs	Count	335	30	365
	%	91.8%	8.2%	100.0%
Grade R; S, 5yrs+; T	Count	769	32	801
	%	96.0%	4.0%	100.0%
Wave 8 Interviewer Age				
less than 40 years	Count	166	29	195
	%	85.1%	14.9%	100.0%
40-49 years	Count	812	85	897
	%	90.5%	9.5%	100.0%
50-59 years	Count	2346	213	2559
	%	91.7%	8.3%	100.0%
60+ years	Count	2107	174	2281
	%	92.4%	7.6%	100.0%
Interviewer Change Indicator				
Same interviewer	Count	3794	287	4081
	%	93.0%	7.0%	100.0%
More than one interviewer	Count	1637	214	1851
	%	88.4%	11.6%	100.0%
Wave 8 Interviewer Sex				
Female	Count	3037	271	3308
	%	91.8%	8.2%	100.0%
Male	Count	2394	230	2624
	%	91.2%	8.8%	100.0%
Wave 8 Interviewer Work History				
Working for another survey agency at time of survey & Done other (phone, market research) survey interviewing	Count	696	55	751
	%	92.7%	7.3%	100.0%
Worked for another survey agency prior to 1st Jan 2006 & Done other (phone, market research) survey interviewing	Count	1440	152	1592
	%	90.5%	9.5%	100.0%
Never worked for another survey agency	Count	2514	230	2744
	%	91.6%	8.4%	100.0%

Working for another survey agency at time of survey & Never done other (phone, market research) survey interviewing	Count	249	16	265
	%	94.0%	6.0%	100.0%
Worked for another survey agency prior to 1st Jan 2006 & Never done other (phone, market research) survey interviewing	Count	532	48	580
	%	91.7%	8.3%	100.0%
Wave 8 Interviewer 'Worry a Lot' Single Item Personality Trait				
1 (Does not apply to me at all)	Count	702	37	739
	%	95.0%	5.0%	100.0%
2, 3	Count	1973	207	2180
	%	90.5%	9.5%	100.0%
4	Count	1197	97	1294
	%	92.5%	7.5%	100.0%
5, 6, 7 (Applies perfectly to me)	Count	1559	160	1719
	%	90.7%	9.3%	100.0%
Wave 8 Interviewer 'Express Myself Easily' Single Item Skills				
1 (Does not apply to me at all), 2, 3	Count	341	21	362
	%	94.2%	5.8%	100.0%
4	Count	703	53	756
	%	93.0%	7.0%	100.0%
5, 6, 7 (Applies perfectly to me)	Count	4387	427	4814
	%	91.1%	8.9%	100.0%
Wave 8 Interviewer 'Can't Help but Look Upset When Things Go Wrong' Single Item Skills				
1 (Does not apply to me at all), 2	Count	1137	82	1219
	%	93.3%	6.7%	100.0%
3, 4, 5	Count	3434	319	3753
	%	91.5%	8.5%	100.0%
6, 7 (Applies perfectly to me)	Count	860	100	960
	%	89.6%	10.4%	100.0%

Table 3: Mean Score and Standard Deviation of the Response Outcome by Area and Interviewer Continuous Variables for the Individual-Level Data

Variable	Statistic	Productive Interview	Refusals
Composite Measure of Negative Area Perception	Mean	20.05	19.73
	Std. Deviation	4.74	4.72
Wave 8 Interviewer Agreeableness Personality Score	Mean	5.69	5.69
	Std. Deviation	0.80	0.82
Wave 8 Interviewer Conscientiousness Personality Score	Mean	5.86	5.86
	Std. Deviation	0.86	0.83
Wave 8 Interviewer Extroversion Personality Score	Mean	4.89	4.74
	Std. Deviation	1.20	1.25
Wave 8 Interviewer Neuroticism Personality Score	Mean	3.04	3.11
	Std. Deviation	1.24	1.13
Wave 8 Interviewer Openness Personality Score	Mean	5.10	5.20
	Std. Deviation	1.04	1.02

Wave 8 Interviewer Unavailability Score	Mean	5.66	5.91
	Std. Deviation	8.66	8.61
Wave 8 Interviewer Unwillingness Score	Mean	5.20	4.61
	Std. Deviation	8.13	7.24
Wave 8 Interviewer Composite Measure of Negative Views on Persuasion of Respondents	Mean	2.74	2.73
	Std. Deviation	0.36	0.32
Wave 8 Interviewer Verbal Communication Skills	Mean	5.22	5.23
	Std. Deviation	0.92	0.88
Wave 8 Interviewer Adaptation Skills	Mean	5.50	5.46
	Std. Deviation	0.84	0.88
Wave 8 Interviewer Connectedness Skills	Mean	5.09	5.05
	Std. Deviation	0.82	0.80
Wave 8 Interviewer Emotional Resilience Skills	Mean	4.10	3.98
	Std. Deviation	1.09	1.09
Wave 8 Interviewer Conformity Skills	Mean	5.40	5.39
	Std. Deviation	0.90	0.90
Wave 8 Interviewer Non-Verbal Communication Skills	Mean	4.99	4.96
	Std. Deviation	1.19	1.17
Wave 8 Interviewer Assertiveness Skills	Mean	4.61	4.57
	Std. Deviation	1.22	1.22
Wave 8 Interviewer Small Talk Skills	Mean	4.22	4.04
	Std. Deviation	1.70	1.68
Wave 8 Interviewer Deliberation Skills	Mean	5.28	5.27
	Std. Deviation	0.88	0.90

Table 4: Frequency Distribution of the Area-Level Variables for Area-Level Data

Category	Frequency	Percent
Government Office Region		
North East	12	8
North West & Merseyside	16	10.7
Yorkshire & Humber	13	8.7
East Midlands	20	13.3
West Midlands	18	12
South West	14	9.3
Eastern	9	6
London	12	8
South East	20	13.3
Wales	5	3.3
Scotland	11	7.3
London Indicator		
Not London	138	92
London	12	8

Table 5: Frequency Distribution of Interviewer-Level Variables for Interviewer-Level Data

**Category**	**Frequency**	**Percent**
Interviewer Grade/ Experience		
A; B	26	9.5
C, 0-4 yrs	107	38.9
C, 5yrs+	45	16.4
D, 0-4 yrs	19	6.9
D, 5yrs+	25	9.1
S, 0-4 yrs	17	6.2
Grade R; S, 5yrs+; T	36	13.1
Interviewer Age		
less than 40 years	10	3.6
40-49 years	45	16.4
50-59 years	117	42.5
60+ years	103	37.5
Interviewer Change Indicator		
Same interviewer	155	56.4
More than one interviewer	120	43.6
Wave 8 Interviewer Sex		
Female	152	55.3
Male	123	44.7
Wave 8 Interviewer Work History		
Working for another survey agency at time of survey & Done other (phone, market research) survey interviewing	37	13.5
Worked for another survey agency prior to 1st Jan 2006 & Done other (phone, market research) survey interviewing	72	26.2
Never worked for another survey agency	130	47.3
Working for another survey agency at time of survey & Never done other (phone, market research) survey interviewing	12	4.4
Worked for another survey agency prior to 1st Jan 2006 & Never done other (phone, market research) survey interviewing	24	8.7
Wave 8 Interviewer 'Worry a Lot' Single Item Personality Trait		
1 (Does not apply to me at all)	35	12.7
2, 3	101	36.7
4	60	21.8
5, 6, 7 (Applies perfectly to me)	79	28.7
Wave 8 Interviewer 'Express Myself Easily' Single Item Personality Trait		
1 (Does not apply to me at all), 2, 3	16	5.8
4	38	13.8
5, 6, 7 (Applies perfectly to me)	221	80.4
Wave 8 Interviewer 'Can't Help but Look Upset When Things Go Wrong' Single Item Personality Trait		
1 (Does not apply to me at all), 2	57	20.7
3, 4, 5	175	63.6
6, 7 (Applies perfectly to me)	43	15.6

Table 6: Mean Score and Standard Deviation for Interviewer-Level Continuous Variables for Interviewer-Level Data

Variable	Statistic	
Wave 8 Interviewer Agreeableness Personality Score	Mean	5.72
	Std. Deviation	0.82
Wave 8 Interviewer Conscientiousness Personality Score	Mean	5.81
	Std. Deviation	0.88
Wave 8 Interviewer Extroversion Personality Score	Mean	4.85
	Std. Deviation	1.20
Wave 8 Interviewer Neuroticism Personality Score	Mean	3.04
	Std. Deviation	1.21
Wave 8 Interviewer Openness Personality Score	Mean	5.16
	Std. Deviation	1.02
Wave 8 Interviewer Unavailability Score	Mean	5.91
	Std. Deviation	8.94
Wave 8 Interviewer Unwillingness Score	Mean	4.92
	Std. Deviation	7.91
Wave 8 Interviewer Composite Measure of Negative Views on Persuasion of Respondents	Mean	2.73
	Std. Deviation	0.34
Wave 8 Interviewer Verbal Communication Skills	Mean	5.08
	Std. Deviation	0.82
Wave 8 Interviewer Adaptation Skills	Mean	5.48
	Std. Deviation	0.85
Wave 8 Interviewer Connectedness Skills	Mean	5.08
	Std. Deviation	0.82
Wave 8 Interviewer Emotional Resilience Skills	Mean	4.07
	Std. Deviation	1.10
Wave 8 Interviewer Conformity Skills	Mean	5.38
	Std. Deviation	0.93
Wave 8 Interviewer Non-Verbal Communication Skills	Mean	5.02
	Std. Deviation	1.18
Wave 8 Interviewer Assertiveness Skills	Mean	4.57
	Std. Deviation	1.23
Wave 8 Interviewer Small Talk Skills	Mean	4.21
	Std. Deviation	1.66
Wave 8 Interviewer Deliberation Skills	Mean	5.30
	Std. Deviation	0.90
